# Supplementary material for: A Molecular Test for Quantifying Functional Notch Signaling Pathway Activity in Human Cancer
Source: Cancers (Basel). 2020 Oct 27;12(11):3142. doi: 10.3390/cancers12113142 (PMC7692325; doi:10.3390/cancers12113142)
Supplement: Supplementary file 1 [file cancers-12-03142-s001.pdf]

cancers

MDPI

Article

A Molecular Test for Quantifying Functional Notch Signaling Pathway Activity in Human Cancer

Kirsten Canté-Barrett [1,†], Laurent Holtzer [2,†], Henk van Ooijen [3], Rico Hagelaar [1], Valentina Cordo' [1], Wim Verhaegh [3], Anja van de Stolpe [2,#] and Jules P. P. Meijerink [1,#,*]

Supplemental Table S1. Scoring of NOTCH target genes with respect to evidence as to direct gene regulation by Notch transcription factors. Evidence score calculation: Response element (1); ChIP (1); Differential expression (down), e.g. by GSI treatment (1); Differential expression (up), e.g. by ICN1 transfection (1); Luciferase assay (1); Cycloheximide addition (1); EMSA (1); Evidence obtained in mouse (0.5 instead of 1).

#	Gene	Evidence + references	Evidence score
1	*CD44*	Response element [1], ChIP[1], GSI treatment [1,2]	3
2	*DTX1*	GSI treatment [3–5], Notch1 knockdown [3], Notch1 induction (mouse) [6]	1.5
3	*EPHB3*	Response element [1,7], ChIP [1,7], GSI treatment [1,7], Luciferase (Depending on cell line) [7], dnMAML induction [7]	4
4	*HES1*	Response element [1,8], ChIP [1,7], GSI treatment [1,5,9–11], dnMAML induction [7], Luciferase [12], Luciferase (mouse) [13], Delta1+CHX (mouse) [14]	4.5
5	*HES4*	Response element [15], ChIP [11], GSI treatment [9,11]	3
6	*HES5*	Response element [8], Notch mutant (mouse) [16], GSI treatment [11,17], NICD transfection (mouse) [18]	2.5
7	*HES7*	GSI treatment [19], Response element (mouse) [20], Luciferase assay (mouse) [20]	2
8	*HEY1*	Response element [8,21], Luciferase (mouse) [22], Cycloheximide (mouse) [23], ChIP [21], GSI treatment [5,11,24]	4
9	*HEY2*	Response element [8], Luciferase (mouse) [18,22], Promoter deletions (mouse) [22], GSI treatment [11], N4ICD overexpression [25], CSL mutation [25], immobilized Dll1 [26] N1ICD transfection (mouse) [18,27], ChIP (mouse) [27], ChIP (human) [28], EMSA [28]	5.5
10	*HEYL*	Response element [8,22], GSI treatment [11], Notch1 knockout (mice) [29], Notch activitation(mouse) [22], Promoter deletion (mouse) [22]	2.5
11	*MYC*	Response element [5], GSI treatment [3,5,9,11], GSI treatment (mouse) [30], Cycloheximide treatment [5], ChIP [3,5,31], ChIP (mouse) [30], EMSA [5], Notch1 knockdown [3]	5
12	*NFKB2*	Response element [32], EMSA [32], luciferase [32], ChIP [33]	4
13	*NOX1*	Response element [1], ChIP [1] , GSI treatment [1]	3
14	*NRARP*	Response element [34], GSI treatment [4,11], Luciferase (mouse) [34], EMSA [34], NICD Mutation (mouse) [35], N3ICD transfection [36]	4.5
15	*PBX1*	Response element [37], Cycloheximide treatment [37], GSI treatment [37], N3ICD inhibition [37,38]	4
16	*PIN1*	Response element [10], Luciferase assay [10], ChIP [10,39], GSI treatment [10], N1ICD overexpression [10]	5
17	*PLXND1*	Response element [40], Luciferase assay [40], dnRBPj [40], N1ICD overexpression [40]	4
18	*SOX9*	Response element [1,41], ChIP [1,41], GSI treatment [1], Notch1 signaling induction [42,43], Cycloheximide treatment [42,43]	5

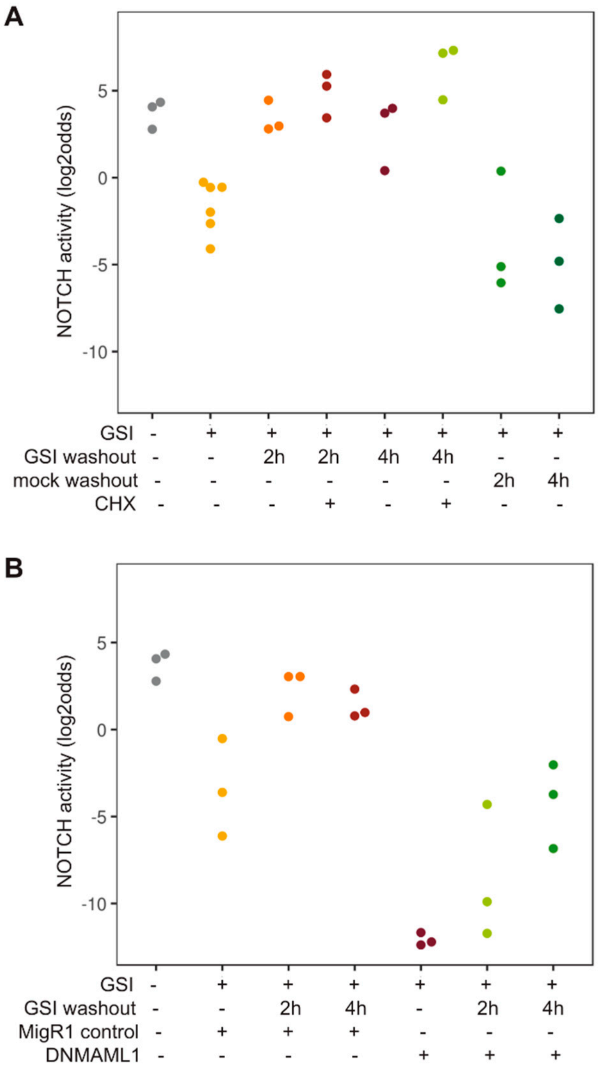

Supplemental Figure S1. Extended validation of the Notch pathway model in CUTLL1 cells. Related to Fig. 2H., dataset GSE29544. CUTLL1 cells subjected to GSI compound E (1μM) for 3 days. (A) From left to right: DMSO control; GSI; GSI followed by 2 hours washout of the GSI; GSI followed by 2 hours washout of the GSI in the presence of cycloheximide (CHX); GSI followed by 4 hours washout of the GSI; GSI followed by 4 hours washout of the GSI in the presence of CHX; GSI with 2 hours mock washout; GSI with 4 hours mock washout. (B) From left to right: DMSO control; GSI in presence of a control viral transcript (MigR1); GSI in the presence of MigR1 with 2 hours washout; GSI in the presence of MigR1 with 4 hours washout; GSI in the presence of a dominant negative viral transcript DNMAML1; GSI in the presence of DNMAML1 with 2 hours washout; GSI in the presence of DNMAML1 with 4 hours washout.

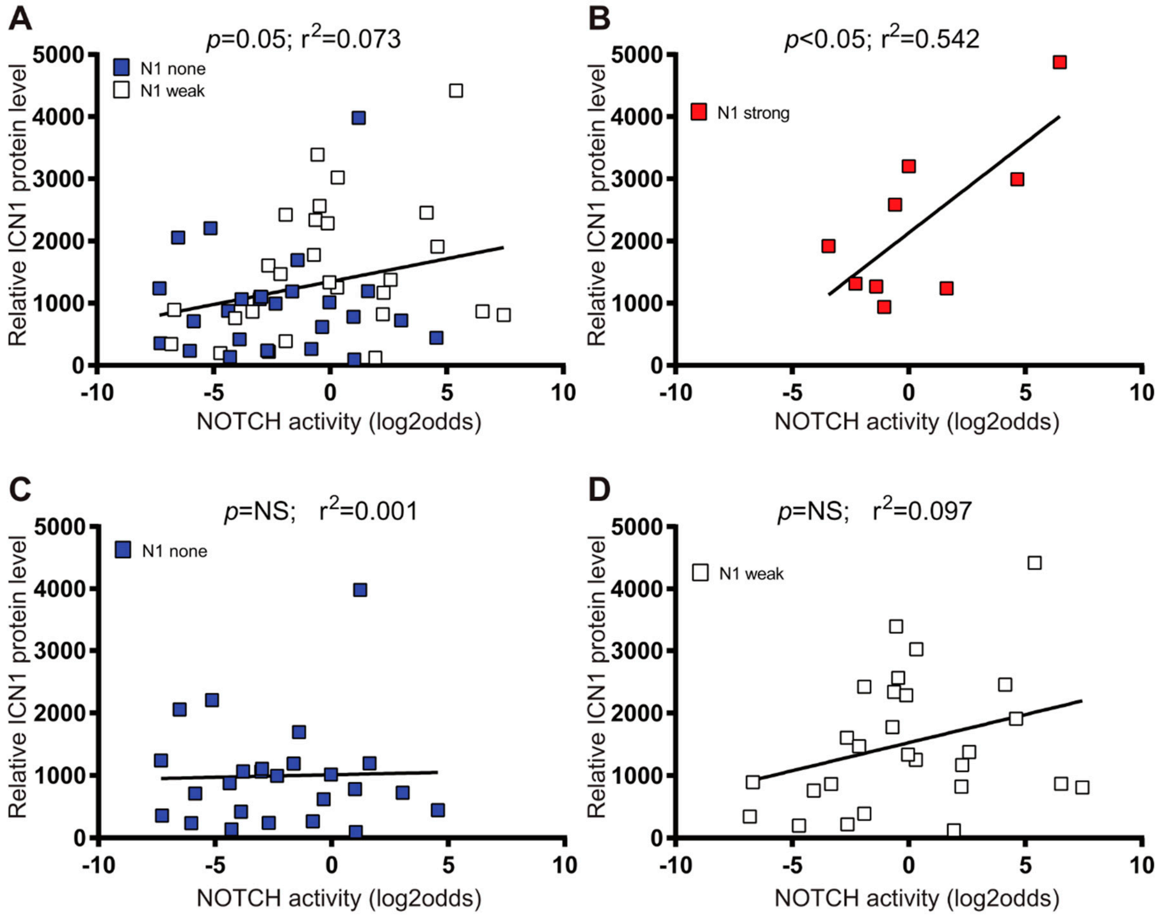

Supplemental Figure S2. Correlations of ICN1 level and Notch pathway activity, divided per NOTCH1-activating mutation status. Related to Fig. 3D. No NOTCH activating mutations (blue symbols), weak NOTCH1 activating mutations (NOTCH1 heterodimerization domain, PEST domain or in FBXW7) (white symbols) and strong NOTCH1 activating mutations (juxtamembrane domain or more than one NOTCH1 activating mutation) (red symbols) are indicated. P-values are indicated. NS: not significant. Correlation of ICN1 protein level and NOTCH pathway activity for samples without and with weak NOTCH1-activating mutations (**A**), with strong NOTCH1-activating mutations (**B**), without NOTCH1-activating mutations (**C**), and with weak NOTCH1-activating mutations (**D**).

References

1. Rodilla, V.; Villanueva, A.; Obrador-Hevia, A.; Robert-Moreno, A.; Fernandez-Majada, V.; Grilli, A.; Lopez-Bigas, N.; Bellora, N.; Alba, M.M.; Torres, F.; et al. Jagged1 is the pathological link between Wnt and Notch pathways in colorectal cancer. *Proc. Natl. Acad. Sci. USA* **2009**, *106*, 6315–6320, doi:10.1073/pnas.0813221106.
2. Kulsum, S.; Raju, N.; Raghavan, N.; Ramanjanappa, R.D.R.; Sharma, A.; Mehta, A.; Kuriakose, M.A.; Suresh, A. Cancer stem cells and fibroblast niche cross talk in an in-vitro oral dysplasia model. *Mol. Carcinog.* **2019**, *58*, 820–831, doi:10.1002/mc.22974.
3. Palomero, T.; Lim, W.K.; Odom, D.T.; Sulis, M.L.; Real, P.J.; Margolin, A.; Barnes, K.C.; O'Neil, J.; Neuberg, D.; Weng, A.P.; et al. NOTCH1 directly regulates c-MYC and activates a feed-forward-loop transcriptional network promoting leukemic cell growth. *Proc. Natl. Acad. Sci. USA* **2006**, *103*, 18261–18266, doi:10.1073/pnas.0606108103.
4. Van de Walle, I.; De Smet, G.; De Smedt, M.; Vandekerckhove, B.; Leclercq, G.; Plum, J.; Taghon, T. An early decrease in Notch activation is required for human TCR-alphabeta lineage differentiation at the expense of TCR-gammadelta T cells. *Blood* **2009**, *113*, 2988–2998, doi:10.1182/blood-2008-06-164871.
5. Weng, A.P.; Millholland, J.M.; Yashiro-Ohtani, Y.; Arcangeli, M.L.; Lau, A.; Wai, C.; Del Bianco, C.; Rodriguez, C.G.; Sai, H.; Tobias, J.; et al. c-Myc is an important direct target of Notch1 in T-cell acute lymphoblastic leukemia/lymphoma. *Genes Dev.* **2006**, *20*, 2096–2109, doi:10.1101/gad.1450406.

6. Deftos, M.L.; He, Y.-W.; Ojala, E.W.; Bevan, M.J. Correlating Notch Signaling with Thymocyte Maturation. *Immunity* **1998**, *9*, 777–786.
7. Jägle, S.; Rönsch, K.; Timme, S.; Andrlová, H.; Bertrand, M.; Jäger, M.; Proske, A.; Schrempp, M.; Yousaf, A.; Michoel, T.; et al. Silencing of the EPHB3 tumor-suppressor gene in human colorectal cancer through decommissioning of a transcriptional enhancer. *Proc. Natl. Acad. Sci.* **2014**, *111*, 4886–4891, doi:10.1073/pnas.1314523111.
8. Katoh, M.; Katoh, M. Integrative genomic analyses on HES/HEY family: Notch-independent HES1, HES3 transcription in undifferentiated ES cells, and Notch-dependent HES1, HES5, HEY1, HEY2, HEYL transcription in fetal tissues, adult tissues, or cancer. *Int. J. Oncol.* **2007**, *31*, 461–466.
9. Palomero, T.; Barnes, K.C.; Real, P.J.; Bender, J.L.G.; Sulis, M.L.; Murty, V.V.; Colovai, A.I.; Balbin, M.; Ferrando, A.A. CUTLL1, a novel human T-cell lymphoma cell line with t(7;9) rearrangement, aberrant NOTCH1 activation and high sensitivity to gamma-secretase inhibitors. *Leukemia* **2006**, *20*, 1279–1287, doi:10.1038/sj.leu.2404258.
10. Rustighi, A.; Tiberi, L.; Soldano, A.; Napoli, M.; Nuciforo, P.; Rosato, A.; Kaplan, F.; Capobianco, A.; Pece, S.; Di Fiore, P.P.; et al. The prolyl-isomerase Pin1 is a Notch1 target that enhances Notch1 activation in cancer. *Nat. Cell Biol.* **2009**, *11*, 133–142, doi:10.1038/ncb1822.
11. Stoeck, A.; Lejnine, S.; Truong, A.; Pan, L.; Wang, H.; Zang, C.; Yuan, J.; Ware, C.; MacLean, J.; Garrett-Engele, P.W.; et al. Discovery of Biomarkers Predictive of GSI Response in Triple-Negative Breast Cancer and Adenoid Cystic Carcinoma. *Cancer Dis.* **2014**, *4*, 1154, doi:10.1158/2159-8290.cd-13-0830.
12. Schroeter, E.H.; Kisslinger, J.A.; Kopan, R. Notch-1 signalling requires ligand-induced proteolytic release of intracellular domain. *Nature* **1998**, *393*, 382–386, doi:10.1038/30756.
13. Jarriault, S.; Brou, C.; Logeat, F.; Schroeter, E.H.; Kopan, R.; Israel, A. Signalling downstream of activated mammalian Notch. *Nature* **1995**, *377*, 355–358, doi:10.1038/377355a0.
14. Kuroda, K.; Tani, S.; Tamura, K.; Minoguchi, S.; Kurooka, H.; Honjo, T. Delta-induced Notch signaling mediated by RBP-J inhibits MyoD expression and myogenesis. *J. Biol. Chem.* **1999**, *274*, 7238–7244.
15. Wang, H.; Zou, J.; Zhao, B.; Johannsen, E.; Ashworth, T.; Wong, H.; Pear, W.S.; Schug, J.; Blacklow, S.C.; Arnett, K.L.; et al. Genome-wide analysis reveals conserved and divergent features of Notch1/RBPJ binding in human and murine T-lymphoblastic leukemia cells. *Proc. Natl. Acad. Sci. USA* **2011**, *108*, 14908–14913, doi:10.1073/pnas.1109023108.
16. de la Pompa, J.L.; Wakeham, A.; Correia, K.M.; Samper, E.; Brown, S.; Aguilera, R.J.; Nakano, T.; Honjo, T.; Mak, T.W.; Rossant, J.; et al. Conservation of the Notch signalling pathway in mammalian neurogenesis. *Development (Cambridge, England)* **1997**, *124*, 1139–1148.
17. Li, G.-H.; Fan, Y.-Z.; Liu, X.-W.; Zhang, B.-F.; Yin, D.-D.; He, F.; Huang, S.-Y.; Kang, Z.-J.; Xu, H.; Liu, Q.; et al. Notch signaling maintains proliferation and survival of the HL60 human promyelocytic leukemia cell line and promotes the phosphorylation of the Rb protein. *Mol. Cell. Biochem.* **2010**, *340*, 7–14.
18. Iso, T.; Chung, G.; Hamamori, Y.; Kedes, L. HERP1 is a cell type-specific primary target of Notch. *J. Biol. Chem.* **2001**, *277*, 6598–6607, doi:10.1074/jbc.M110495200.
19. Diaz-Cuadros, M.; Wagner, D.E.; Budjan, C.; Hubaud, A.; Tarazona, O.A.; Donelly, S.; Michaut, A.; Al Tanoury, Z.; Yoshioka-Kobayashi, K.; Niino, Y.; et al. In vitro characterization of the human segmentation clock. *Nature* **2020**, *580*, 113–118, doi:10.1038/s41586-019-1885-9.
20. Bessho, Y.; Miyoshi, G.; Sakata, R.; Kageyama, R. Hes7: A bHLH-type repressor gene regulated by Notch and expressed in the presomitic mesoderm. *Genes Cells* **2001**, *6*, 175–185.
21. Hamidi, H.; Gustafason, D.; Pellegrini, M.; Gasson, J. Identification of novel targets of CSL-dependent Notch signaling in hematopoiesis. *PLoS ONE* **2011**, *6*, e20022.
22. Maier, M.M.; Gessler, M. Comparative analysis of the human and mouse Hey1 promoter: Hey genes are new Notch target genes. *Biochem. Biophys. Res. Commun.* **2000**, *275*, 652–660, doi:10.1006/bbrc.2000.3354.
23. Iso, T.; Sartorelli, V.; Chung, G.; Shichinohe, T.; Kedes, L.; Hamamori, Y. HERP, a new primary target of Notch regulated by ligand binding. *Mol. Cell. Biol.* **2001**, *21*, 6071–6079.
24. Alaña, L.; Sesé, M.; Cánovas, V.; Punyal, Y.; Fernández, Y.; Abasolo, I.; de Torres, I.; Ruiz, C.; Espinosa, L.; Bigas, A.; et al. Prostate tumor OVerexpressed-1 (PTOV1) down-regulates HES1 and HEY1 notch targets genes and promotes prostate cancer progression. *Mol. Cancer* **2014**, *13*, 74.
25. Rad, E.B.; Hammerlindl, H.; Wels, C.; Popper, U.; Menon, D.R.; Breiteneder, H.; Kitzwoegerer, M.; Hafner, C.; Herlyn, M.; Bergler, H.; et al. Notch4 Signaling Induces a Mesenchymal-Epithelial-like Transition in Melanoma Cells to Suppress Malignant Behaviors. *Cancer Res.* **2016**, *76*, 1690–1697.

26. Kim, H.; Huang, L.; Critser, P.J.; Yang, Z.; Chan, R.J.; Wang, L.; Carlesso, N.; Voytik-Harbin, S.L.; Bernstein, I.D.; Yoder, M.C. Notch ligand Delta-like 1 promotes in vivo vasculogenesis in human cord blood-derived endothelial colony forming cells. *Cytotherapy* **2015**, *17*, 579–592.
27. Li, Y.; Hibbs, M.A.; Gard, A.L.; Shylo, N.A.; Yun, K. Genome-wide analysis of N1ICD/RBPJ targets in vivo reveals direct transcriptional regulation of Wnt, SHH, and hippo pathway effectors by Notch1. *STEM CELLS* **2012**, *30*, 741–752.
28. Kulic, I.; Robertson, G.; Chang, L.; Baker, J.H.E.; Lockwood, W.W.; Mok, W.; Fuller, M.; Fournier, M.; Wong, N.; Chou, V.; et al. Loss of the Notch effector RBPJ promotes tumorigenesis. *J. Exp. Med.* **2014**, *212*, 37–52.
29. Leimeister, C.; Schumacher, N.; Steidl, C.; Gessler, M. Analysis of HeyL expression in wild-type and Notch pathway mutant mouse embryos. *Mech. Dev.* **2000**, *98*, 175–178.
30. Sharma, V.M.; Calvo, J.A.; Draheim, K.M.; Cunningham, L.A.; Hermance, N.; Beverly, L.; Krishnamoorthy, V.; Bhasin, M.; Capobianco, A.J.; Kelliher, M.A. Notch1 contributes to mouse T-cell leukemia by directly inducing the expression of c-myc. *Mol. Cell. Biol.* **2006**, *26*, 8022–8031, doi:10.1128/MCB.01091-06.
31. Liao, W.-R.; Hsieh, R.-H.; Hsu, K.-W.; Wu, M.-Z.; Tseng, M.-J.; Mai, R.-T.; Lee, Y.-H.W.; Yeh, T.-S. The CBF1-independent Notch1 signal pathway activates human c-myc expression partially via transcription factor YY1. *Carcinogenesis* **2007**, *28*, 1867–1876.
32. Oswald, F.; Liptay, S.; Adler, G.; Schmid, R.M. NF-kappaB2 is a putative target gene of activated Notch-1 via RBP-Jkappa. *Mol. Cell. Biol.* **1998**, *18*, 2077–2088.
33. Vilimas, T.; Mascarenhas, J.; Palomero, T.; Mandal, M.; Buonamici, S.; Meng, F.; Thompson, B.; Spaulding, C.; Macaroun, S.; Alegre, M.-L.; et al. Targeting the NF-kappaB signaling pathway in Notch1-induced T-cell leukemia. *Nat. Med.* **2006**, *13*, 70–77, doi:10.1038/nm1524.
34. Pirot, P.; van Grunsven, L.A.; Marine, J.-C.; Huylebroeck, D.; Bellefroid, E.J. Direct regulation of the Nrarp gene promoter by the Notch signaling pathway. *Biochem. Biophys. Res. Commun.* **2004**, *322*, 526–534, doi:10.1016/j.bbrc.2004.07.157.
35. Krebs, L.T.; Deftos, M.L.; Bevan, M.J.; Gridley, T. The Nrarp gene encodes an ankyrin-repeat protein that is transcriptionally regulated by the notch signaling pathway. *Dev. Biol.* **2002**, *238*, 110–119.
36. Xiao, Y.; Ye, Y.; Zou, X.; Jones, S.; Yearsley, K.; Shetuni, B.; Tellez, J.; Barsky, S.H. The lymphovascular embolus of inflammatory breast cancer exhibits a Notch 3 addiction. *Oncogene* **2010**, *30*, 287–300.
37. Park, J.T.; Shih, I.-M.; Wang, T.-L. Identification of Pbx1, a potential oncogene, as a Notch3 target gene in ovarian cancer. *Cancer Res.* **2008**, *68*, 8852–8860, doi:10.1158/0008-5472.CAN-08-0517.
38. Magnani, L.; Stoeck, A.; Zhang, X.; Lánczky, A.; Mirabella, A.C.; Wang, T.-L.; Gyorffy, B.; Lupien, M. Genome-wide reprogramming of the chromatin landscape underlies endocrine therapy resistance in breast cancer. *Pro. Natl. Acad. Sci.* **2013**, *110*, E1490-1499, doi:10.1073/pnas.1219992110.
39. Chen, X.; Thiaville, M.M.; Chen, L.; Stoeck, A.; Xuan, J.; Gao, M.; Shih, I.-M.; Wang, T.-L. Defining NOTCH3 target genes in ovarian cancer. *Cancer Res.* **2012**, *72*, 2294–2303, doi:10.1158/0008-5472.CAN-11-2181.
40. Rehman, M.; Gurrapu, S.; Cagnoni, G.; Capparuccia, L.; Tamagnone, L. PlexinD1 Is a Novel Transcriptional Target and Effector of Notch Signaling in Cancer Cells. *PLoS ONE* **2016**, *11*, e0164660, doi:10.1371/journal.pone.0164660.
41. Zong, Y.; Panikkar, A.; Xu, J.; Antoniou, A.; Raynaud, P.; Lemaigre, F.; Stanger, B.Z. Notch signaling controls liver development by regulating biliary differentiation. *Development* **2009**, *136*, 1727–1739, doi:10.1242/dev.029140.
42. Martini, S.; Bernoth, K.; Main, H.; Ortega, G.D.C.; Lendahl, U.; Just, U.; Schwanbeck, R. A critical role for Sox9 in notch-induced astrogliogenesis and stem cell maintenance. *STEM CELLS* **2013**, *31*, 741–751, doi:10.1002/stem.1320.
43. Meier-Stiegen, F.; Schwanbeck, R.; Bernoth, K.; Martini, S.; Hieronymus, T.; Ruau, D.; Zenke, M.; Just, U. Activated Notch1 target genes during embryonic cell differentiation depend on the cellular context and include lineage determinants and inhibitors. *PLoS ONE* **2010**, *5*, e11481, doi:10.1371/journal.pone.0011481.

© 2020 by the authors. Licensee MDPI, Basel, Switzerland. This article is an open access article distributed under the terms and conditions of the Creative Commons Attribution (CC BY) license (http://creativecommons.org/licenses/by/4.0/).
